# Fatty acid composition of diets of early school-age children and its health implications

**DOI:** 10.12669/pjms.316.7614

**Published:** 2015

**Authors:** Małgorzata Kostecka

**Affiliations:** 1 Małgorzata Kostecka, University of Life Sciences in Lublin, Faculty of Food Science and Biotechnology, Ul. Akademicka 15, 20-950 Lublin, Poland

**Keywords:** Fatty acids, PUFA, MUFA, SFA, Children

## Abstract

**Objective:**

The main objective of this study was to determine the amount and type of fat consumed by early school-age children per day. Dietary fat intake as a percentage of the total calorie intake was also estimated.

**Methods:**

The study was conducted in Lublin, the largest city in south-east Poland, between January 2014 and April 2014, on 702 randomly selected children, i.e. 3% of the total population of early school-age children in the research area. The parents were asked to provide information about the type and amount of food consumed by their children daily. A standard food frequency questionnaire was used.

**Results:**

Dietary levels of saturated fat were elevated in the analyzed populations and were the source of 13.33% of daily calorie intake. In the studied population, the ratio of saturated to monounsaturated and polyunsaturated fatty acids was determined at 3.25: 2.95: 1. Dietary intake of α-linolenic acid (ALA) reached 1.057 ± 0.55 g (0.63% of daily calorie intake) on average, and dietary intake of linoleic acid (LA) was determined at 3.86 ± 2.51 g (2.31% of daily calorie intake).

**Conclusions:**

The average total calorie intake of children aged 6-11 years was 1445.66 calories per day. Average fat intake was 29.64 % of the total calorie intake. The highest intake of SFAs was found in the youngest age group of 6- to 8-year-olds. The type of food consumed by children affected the amount and type of dietary fat in all age groups.

## INTRODUCTION

Nutrition and a balanced diet are the key factors that influence healthy growth and development of children, next to physical activity. The ratio of saturated to monounsaturated and polyunsaturated fatty acids in the diet plays a major role in the development of life-style diseases.^1^ The amount of energy provided by dietary fats should be kept within a healthy limit, and children’s diets should provide 30% of energy from fat. Diets should be rich in omega-3 and omega-6 fatty acids, but low in trans fats.^2^,^3^ The daily intake of omega-3 fatty acids should range from 0.5 to 2% of total calories consumed.^4^ Essential fatty acid deficiencies are believed to affect the maturation of the central nervous system, and fat restriction may impede growth and deprive children of nutrients such as fat-soluble vitamins. Parents who wish to restrict fat intake should be warned of the potential for delayed growth when fat intake is less than 20 percent of calories 2,5

According to the recommendations of the European Food Safety Authority (EFSA), the American Institute of Medicine (AIM) and nutrition standards for the Polish population, 4-8% of the total energy intake of children older than 3 years should be derived from omega-6 fatty acids. The recommended daily intake of omega-3 fatty acids is estimated at 2 g of α-linolenic acid and 200-250 mg of long-chain polyunsaturated fatty acids (LC-PUFAs).^6^-^8^

According to the 2010 Dietary Guidelines Report, approximately 35% of the calories in kids’ diets come from SOFAS (solid fats and added sugars) in foods such as cakes, cookies, granola bars, soda and pizza when it really should be more like 10%.^9^,^10^

The main objective of this study was to determine the amount and type of fat consumed by early school-age children per day. Dietary fat intake as a percentage of the total calorie intake was also estimated. There is a scarcity of published research on the quantitative and qualitative fat intake in children between the ages of 6 and 11 years.

## METHODS

The study involved children aged 6-11 years because primary school students have greater freedom in their food choices than younger children, and are more likely to decide not only about the quantity but also about the quality of food consumed. At this age, peer groups begin to influence children’s food choices and eating habits.

The study was conducted in Lublin, the largest city in south-east Poland, between January 2014 and April 2014, on 702 randomly selected children, i.e. 3% of the total population of early school-age children in the research area. In the first stage, parents or legal guardians provided informed consent for their children to participate in this research study, including anthropometric measurements. Children with diabetes, cystic fibrosis, chronic bowel disease and other conditions that require a fat-restricted diet were excluded from the study, which resulted in an enrollment of 335 primary school students. All parents and guardians were asked to fill out a short questionnaire to provide information about the family’s eating habits, meal preparation methods, and knowledge about child nutrition. Closed-ended questions and randomization of response items were used to minimize the impact of the so-called first response. Parents and guardians were asked about the type and amount of food consumed by children daily and they kept daily food records for a week. Portion size was quantified with the use of household measures. A standard food frequency questionnaire was used. The surveyed children were also subjected to anthropometric (height and weight) measurements, and the results were used to calculate the Cole index and assess the children’s nutritional status.

Dietary recalls were processed and evaluated in the Diet 5 computer program developed by the National Food and Nutrition Institute in Warsaw based on Polish food composition and nutrition tables.^11^ A computer program was used to calculate the total daily calorie intake per child, the percentage of calories from fat, and the type of dietary fat consumed (saturated, unsaturated). The percentages of SFAs, MUFAs and PUFAs in the daily diets of children werealso determined based on the intake of individual fatty acids. The results were processed statistically by Student’s t-test in Excel at the 95% confidence level.

## RESULTS

The surveyed children were aged 6-11 years (average age 9.1 ± 2.3 years), and they attended public primary schools. Girls accounted for 54% of the surveyed population, and 9-year-olds were the dominant age group. It has been estimated that 17.1% of girls and 13.6% of boys in the studied population were overweight or obese, and 8.8% of girls and 5.2% of boys were characterized by low body weight and energy deficiency.

The average calorie content of the menus surveyed was 1445.66±207.4 kcal, and the average percentage of energy from fat reached 29.64±7.12. In the analyzed population, the most commonly consumed fats were butter (88% of children), soft margarine (26%), vegetable oils (46%), lard and palm oil (12% of parents reported the use of these types of fat for frying).

**Fig.1:**
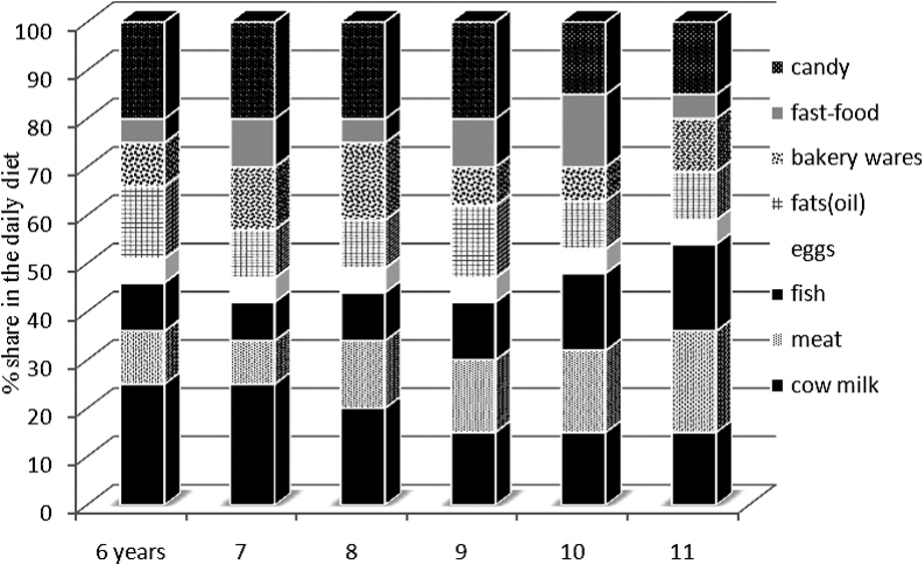
The frequency [%] of intake of foods rich in fatty acids, depending on the age of children.

Daily calorie intake and the amount of fat in the diet varied across age groups. The above could be attributed to differences in eating habits and ingredients selected for meal preparation in the surveyed families. However, the percentage of calories coming from fat was not directly correlated with the percentage content of SFAs (saturated fat) in the diet(Table-I). Children whose diets were characterized by above-average amounts of SFAs consumed significantly more cow’s milk (p<0.05), hard cheese (p<0.05), potato chips and French fries (p<0.05), and bakery wares. Children whose diets were relatively low in SFAs consumed mostly poultry meat (p<0.05), poultry cold meats (p<0.05) and sweets such as chocolate and candy (p<0.05) (Fig.1). Meat intake levels were too high in bothgroups, at 2-3 servings per day. PUFAs had the highest share of the diet in the oldest children who consumed significantly more fish and less sweets.

**Table-I:** Calorie intake and share of fat in the diets of children from the city of Lublin.

Age of children	Total calorie intake [kcal]	% of calorie intake as fat	%of saturated fat	%of unsaturated fat	
				MUFAs	PUFAs
>6-8 years	1500.4±221.5	30.83	14.04	13.36	3.73
>9-10 years	1424.0±206.1	26.55	12.52	10.46	3.56
>11 years	1413.6±194.5	31.55	13.45	13.0	5.09

**Fig.2:**
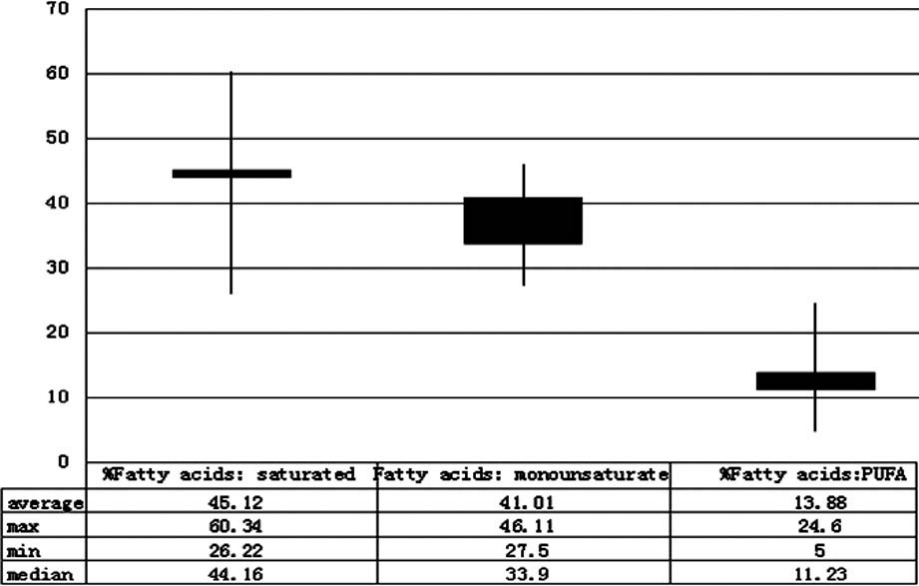
Percentages of saturated, monounsaturated and polyunsaturated fatty acids in the daily diets of children.

The energy derived from saturated fats should not exceed 10% of daily calorie intake because saturated fats influence the serum concentrations of total cholesterol and low-density lipoproteins (LDL). Dietary levels of saturated fat were elevated in the analyzed populations and were the source of 13.33% of daily calorie intake. In the studied population (Fig.2), the ratio of saturated to monounsaturated and polyunsaturated fatty acids was determined at 3.25: 2.95: 1.

The average intake of linoleic acid (LA) was determined at 3.86 ± 2.51 g (2.31% of daily calorie intake), and the main dietary source of linoleic acid was rapeseed oil which is popularly used for frying and in salads. In this study, diets characterized by high levels of linoleic acid contained also milk, dairy products, walnuts and linseed oil. The dietary intake of α-linolenic acid (ALA) was determined at 1.057 ± 0.55 g (0.63% of daily calorie intake) on average (Table-II).

**Table-II:** Estimated daily intake of fatty acids in the diet of children from the city of Lublin.

	Age of children			Mean value in group
	>6-8 years	>8-10 years	>10-11 years	
Total SFAs (g/day)	23.52	24.3	20.91	22.91±8.37
Total MUFAs (g/day)	23.1	18.43	20.87	20.80±7.79
Total PUFAs (g/day)	5.95	6.85	7.15	6.65±2.98
P:S ratio	0.25	0.28	0.34	0.29
18:2(g/day)	3.54	3.95	4.09	3.86±2.51
20:4(g/day)	0.064	0.075	0.077	0.072±0.06
20:5(g/day)	0.0083	0.0086	0.0088	0.0085±0.004
18:3(g/day)	1.049	1.048	1.074	1.057±0.55
22:6(g/day)	0.0313	0.0261	0.0401	0.0325±0.024
Total n-6 PUFAs (g/day)	3.604	4.025	4.167	3.932
Total n-3 PUFAs (g/day)	1.0803	1.0741	1.1141	1.0895
n-6:n-3 PUFA ratio	3.3	3.8	3.7	3.6
18:2(mg/kg body wt)	144.7	157.9	188.4	139.1-203
18:3(mg/kg body wt)	40.9	40.3	50.3	38.1-55.6
20:4(mg/kg body wt)	2.97	3.32	3.45	2.59-3.9
22:6(mg/kg body wt)	1.39	1.23	1.54	1.17-1.72

SFAs –saturated fatty acids, MUFAs- monounsaturated fatty acids, PUFAs-polyunsaturated fatty acids; P:S - ratio of polyunsaturated to saturated fatty acids.

## DISCUSSION

Total fat intake in the studied 10-11-year-old children exceeded the recommendations and should be lowered. In the other age groups, the amount of fat in the diet was consistent with the recommendations. In comparison with dietary fat intake in children from other European countries, the average fat intake determined in this study (29.64 % of the total calorie intake) was within the reference range. The percentage of energy coming from fat was highest in children from Greece^12^, including Crete^13^, and Spain^14^, and it exceeded 40%. In Europe, only the diets of Belgian children met the recommended fat intakes.^15^

In the present study, the fatty acid composition of children’s diets was also undesirable. In all age groups, the dietary intake of SFAs was higher than the recommended limit by approximately 2.5-4.0%, regardless of gender. Similar results were reported by Merkiel^16^ in a study of Polish 6-year-old children. MUFAs and PUFAs had a similar share of the daily diet in Polish children from different age groups^16^, but it was much lower than in the diets of children living in the Mediterranean countries. Cretan^13^, Portuguese^17^ and Spanish^18^ children consume more PUFAs daily than children in Poland. Due to the high proportion of dairy products, confectionery and meat products, SFAs are the predominant fatty acid group in the diets of Polish and British children, which is reflected in the PUFA:SFA ratio determined at approximately 0.3.^19^

Linoleic acid (LA) and α-linolenic (ALA) play a major role in the growth and development of infants and small children. The minimum intake levels for essential fatty acids to prevent deficiency symptoms have been estimated at 2.5%E LA and 0.5%E ALA. Based on epidemiological studies and randomized controlled trials of coronary heart disease (CHD) events, the minimum recommended level of total PUFA consumption for lowering LDL and total cholesterol concentrations, increasing HDL cholesterol concentrations and decreasing the risk of CHD events is 6%E.^20^ In this study, PUFAs had a low share of the daily diet (3.99%).

Based on the biological significance of omega-3 and omega-6 fatty acids, their optimal ratio was determined at 4-5: 1. In the studied children’s diets, the ratio of omega-6 to omega-3 fatty acids was satisfactory (3.6:1) in all age groups, which could exert a protective effect on the heart and prevent inflammations in childhood and adulthood.^21^

In the analyzed group of children, LC-PUFAs EPA and DHA had a too low share of the daily diet. The dietary intake of DHA and EPA per kg body weight was 15-fold and up to 24-fold lower than the recommended levels, respectively.^19^,^22^ The intake of linoleic acid per kg body weight was 2-fold lower than the recommended value. In contrast, the daily intake of α-linolenic acid was consistent with the recommendations.

In studies conducted by other authors, fish and seafood products were the largest contributors to DHA (76%) and EPA (59%) intake, whereas meat, including poultry and game meat, provided only small amounts of those fatty acids. Meat consumption was 8.5 times greater than fish/seafood consumption. Australian children do not consume the recommended amounts of long-chain omega-3 fatty acids, in particular DHA, which could be due to low fish consumption.^23^

## CONCLUSIONS

The average total calorie intake of children aged 6-11 years was 1445.66 calories per day. Average fat intake was 29.64 % of the total calorie intake, and SFAs accounted for 13.33%. The highest intake of SFAs was found in the youngest age group of 6- to 8-year-olds. The PUFA: SFA ratio in this group was lowest at 0.25, compared with the average of 0.29 in the entire population. The amount of EPA and DHA in the diet was several times lower than the recommended levels, but the ratio of omega-6 to omega-3 fatty acids was consistent with the recommendations (3.6:1 on average). The type of food consumed by children affected the amount and type of dietary fat in all age groups. SFA concentrations depended mainly on the amount of bakery products, milk and dairy products consumed by children. In contrast, PUFA levels were affected primarily by the frequency of fish consumption, which was highest in the age group of 10-11-year-olds.
